# Permanent draft genome sequence of *Frankia* sp. NRRL B-16219 reveals the presence of canonical *nod* genes, which are highly homologous to those detected in *Candidatus* Frankia Dg1 genome

**DOI:** 10.1186/s40793-017-0261-3

**Published:** 2017-09-04

**Authors:** Amir Ktari, Imen Nouioui, Teal Furnholm, Erik Swanson, Faten Ghodhbane-Gtari, Louis S. Tisa, Maher Gtari

**Affiliations:** 1 Laboratoire Microorganismes et Biomolécules Actives, Université Tunis El Manar (FST) & Université de Carthage (INSAT), 2092 Tunis, Tunisia; 2 Department of Molecular, Cellular, and Biomedical Sciences, University of New Hampshire, 289 Rudman Hall, 46 college Road, Durham, NH 03824-2617 USA

**Keywords:** *Frankia*, Actinorhizal symbiosis, Plant-microbe interactions, Genome, Canonical nod genes, *Ceanothus*

## Abstract

*Frankia* sp. NRRL B-16219 was directly isolated from a soil sample obtained from the rhizosphere of *Ceanothus jepsonii* growing in the USA. Its host plant range includes members of *Elaeagnaceae* species. Phylogenetically, strain NRRL B-16219 is closely related to *“Frankia discariae*” with a 16S rRNA gene similarity of 99.78%. Because of the lack of genetic tools for *Frankia*, our understanding of the bacterial signals involved during the plant infection process and the development of actinorhizal root nodules is very limited. Since the first three *Frankia* genomes were sequenced, additional genome sequences covering more diverse strains have helped provide insight into the depth of the pangenome and attempts to identify bacterial signaling molecules like the rhizobial canonical *nod* genes. The genome sequence of *Frankia* sp. strain NRRL B-16219 was generated and assembled into 289 contigs containing 8,032,739 bp with 71.7% GC content. Annotation of the genome identified 6211 protein-coding genes, 561 pseudogenes, 1758 hypothetical proteins and 53 RNA genes including 4 rRNA genes. The NRRL B-16219 draft genome contained genes homologous to the rhizobial common nodulation genes clustered in two areas. The first cluster contains *nod*ACIJH genes whereas the second has *nod*AB and *nod*H genes in the upstream region. Phylogenetic analysis shows that *Frankia nod* genes are more deeply rooted than their sister groups from rhizobia. PCR-sequencing suggested the widespread occurrence of highly homologous *nod*A and *nod*B genes in microsymbionts of field collected *Ceanothus americanus*.

**Electronic supplementary material:**

The online version of this article (10.1186/s40793-017-0261-3) contains supplementary material, which is available to authorized users.

## Introduction

The symbiosis resulting from members of the genus 10.1601/nm.7635 interacting with the roots of 8 dicotyledonous plant families (referred to actinorhizal plants) is found worldwide and contributes to the ability of actinorhizal pioneer plants to grow in poor and marginally fertile soils [1]. This symbiotic association has drawn interest because of its higher rate of soil nitrogen input and the ability of the plants to overcome harsh environmental conditions [2]. The molecular mechanism for the establishment of an actinorhizal nitrogen-fixing root nodule remains elusive [3]. Molecular phylogeny of the 10.1601/nm.7635 genus has consistently identified four main clusters regardless of the typing locus used [1]. These 10.1601/nm.7635 clusters also follow and support the host specificity groups proposed by Baker [4]. Cluster 1 is divided into sub-cluster 1a including 10.1601/nm.7636 and relatives that are infective on *Alnus* and *Myricaceae* and sub-cluster 1b strains that are infective on *Allocasuarina*, *Casuarina* and *Myricaceae* including 10.1601/nm.29311 [5]. Cluster 2 contains 10.1601/nm.29887 [6] and uncultured microsymbionts of *Coriariaceae*, *Datiscaceae*, *Dryadoideae* and *Ceanothus*, while cluster 3, associated 10.1601/nm.29312 [5], *“* 10.1601/nm.30356 *”* [7] and closely related strains are infective on *Colletieae*, *Elaeagnaceae*, *Gymnostoma* and *Myricaceae*. Finally, cluster 4 groups a broad range of non-nitrogen-fixing and infective strains including 10.1601/nm.29990 species [8] together with “*F. asymbiotica*” [9] and other related strains that are unable to establish a symbiosis with actinorhizal plants. As has been established for rhizobial and arbuscular mycorrhizal symbioes, the LysM-RLKs are also involved in the perception of 10.1601/nm.7635 signal molecules by the actinorhizal plant [10, 11]. However, the bacterial signals triggering this symbiosis remain unknown. At present, more than 30 10.1601/nm.7635 genomes from strains in pure culture have been sequenced and annotated [12–30] and two Candidatus genomes were generated from nodule metagenomes [31, 32]. Analysis of the 10.1601/nm.7635 genomes failed to reveal the presence of common canonical *nodABC* genes [33] which also appear to be missing in several photosynthetic [34] and non-photosynthetic [35] bradyrhizobia. The only exceptions were found in the two *Candidatus Frankia* genomes, which contained the canonical *nod*ABC and sulfotransferase *nod*H genes [32, 36]. This contradictory situation justifies additional sequencing of genomes from cultivated 10.1601/nm.7635 strains to gain insight into the depth of the pangenome pool covered. Here we report the first proof of the presence of rhizobial homologous canonical *nodABCH* genes within the draft genome of cultivated 10.1601/nm.7635 isolate, strain 10.1601/strainfinder?urlappend=%3Fid%3DNRRL+B-16219 and widespread occurrence of *nodAB* in field collected *Ceanothus americanus* microsymbionts.

## Organism information

### Classification and features

**Fig. 1:**
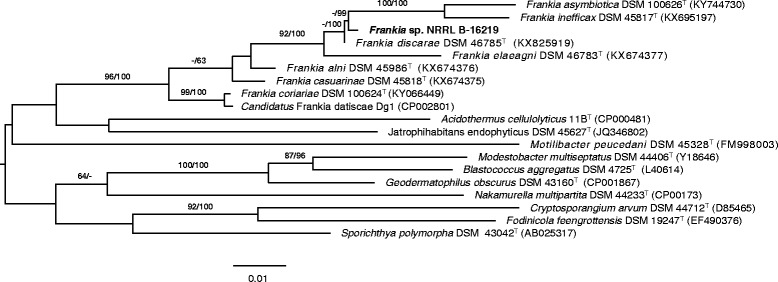
Maximum likelihood (ML) phylogenetic tree based on the 16S rRNA gene sequences (1400 nt), showing the relationships between *Frankia* NRRL B-16219 and *Frankia* species. The ML tree was inferred using the GTR + GAMMA model and rooted by midpoint-rooting; the branches are scaled in terms of the expected number of substitutions per site. The numbers *above* the branches are support values when larger than 60% from ML (*left*) and MP (*right*) bootstrapping

Strain 10.1601/strainfinder?urlappend=%3Fid%3DNRRL+B-16219 metabolizes short fatty acids, TCA-cycle intermediates and carbohydrates (Table 1). It is infective on members of *Elaeagnaceae* and *Morella cerifera* and produces effective root nodules [4, 37]. In coherence with its host range, strain 10.1601/strainfinder?urlappend=%3Fid%3DNRRL+B-16219 is phylogenetically affiliated to cluster 3, known to effectively nodulate members of *Elaeagnaceae*, *Rhamnaceae* and *Myricaceae* families. Phylogenetic analysis based on 16S rRNA gene sequence showed that strain 10.1601/strainfinder?urlappend=%3Fid%3DNRRL+B-16219 was most closely related to type strains of *“* 10.1601/nm.30356 *”* 10.1601/strainfinder?urlappend=%3Fid%3DDSM+46785 ^T^ (99.78%) and 10.1601/nm.29312 (98.26%) (Fig. 1).

**Table 1:** Classification and general features of *Frankia* sp. strain NRRL B-16219 according to MIGS [45]

MIGS ID	Property	Term	Evidence code^a^
	Classification	Domain *Bacteria*	TAS [46]
		Phylum *Actinobacteria*	TAS [47]
		Class *Actinobacteria*	TAS [48]
		Order *Frankiales*	TAS [49]
		Family *Frankiaceae*	TAS [50, 51]
		Genus *Frankia*	TAS [52, 53]
		Species *Frankia sp*.	IDR
		Strain NRRL B-16219IDA	
	Gram stain	Positive	IDA
	Cell shape	Filament-shaped	IDA
	Motility	Non-motile	NAS
	Sporulation	Sporulating	NAS
	Temperature range	25–35 °C	TAS [5]
	Optimum temperature	28 °C	TAS [5]
	pH range; Optimum	pH 6.3 – pH 6.8	NAS
	Carbon source	short fatty acids, TCA-cycle intermediates and carbohydrates	IDA
MIGS-6	Habitat	Soil and Host-associated	IDA
MIGS-6.3	Salinity	Not reported	
MIGS-22	Oxygen requirement	Aerobic	NAS
MIGS-15	Biotic relationship	Free-living and Host plant-associated	NAS
MIGS-14	Pathogenicity	Non-pathogen	NAS
MIGS-4	Geographic location	Soil beneath *Ceanothus jepsonii*, USA	IDA
MIGS-5	Sample collection	1982	IDA
MIGS-4.1	Latitude	Not reported	-
MIGS-4.2	Longitude	Not reported	-
MIGS-4.4	Altitude	Not reported	-

^a^ Evidence codes – *IDA* INFERRED FROM DIRECT ASSAY, *TAS* traceable author statement (i.e., a direct report exists in the literature) *NAS* non-traceable author statement (i.e., not directly observed for the living, isolated sample, but based on a generally accepted property for the species, or anecdotal evidence)

**Fig. 2:**
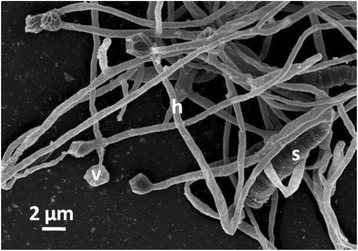
Scanning electron micrograph of strain NRRL B-16219 after growth for 4 weeks in liquid DPM medium at 28 °C showing hyphae (h), vesicles (v) and sporangia (s)

10.1601/nm.7635 sp. strain 10.1601/strainfinder?urlappend=%3Fid%3DNRRL+B-16219 shows typical 10.1601/nm.7635 morphological structures; branched hyphae, vesicles, the site of nitrogenase activity, and multilocular sporangia containing non-motile spores (Fig. 2).

**Fig. 3:**
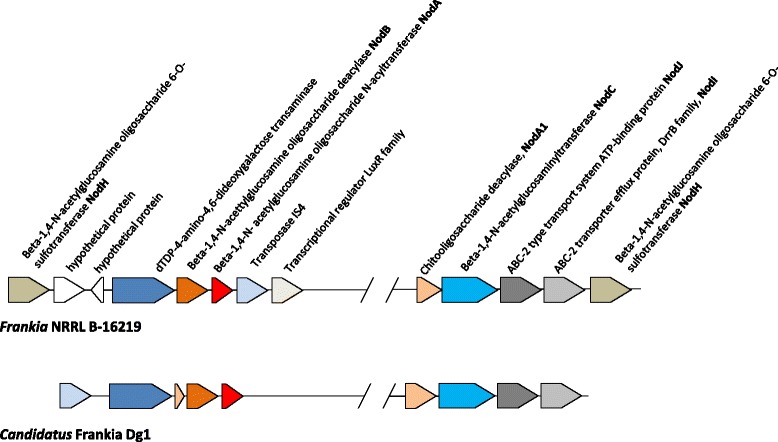
Organization of *nod* genes in *Frankia* NRRL B-16219 and *Candidatus* Frankia datiscae Dg1 genomes. Sizes, localization and orientation of the genes are displayed proportionally. These genes are not detectable in any other *Frankia* genome except *Candidatus* Frankia Dg2

#### Extended feature descriptions

Strain 10.1601/strainfinder?urlappend=%3Fid%3DNRRL+B-16219 represents one of the rare 10.1601/nm.7635 strains directly isolated from soil on plate medium without passing through plant trapping assay. The strain was isolated from the rhizosphere of *Ceanothus jepsonii* [37] following a complex protocol of soil treatment with phenol (0.7%), sample fractionation through ultracentrifugation in sucrose density gradient, and plating on solid DPM without nitrogen source. Strain 10.1601/strainfinder?urlappend=%3Fid%3DNRRL+B-16219 developed unpigmented white colonies after 4 weeks growth on DPM medium at 28 °C without shaking. The strain was phenotyped using GENIII microplates in an Omnilog device (BIOLOG Inc., Haywood, USA) as previously described [5]. It was able to metabolize acetic acid, citric acid, D-cellobiose, dextrin, D-fructose, D-mannitol, D-mannose, fructose-6-phosphate, fusidic acid, glucose-6-phosphate, D and L malic acid, *p*-hydroxy-phenylacetic acid, propionic acid and D-serine and to grow in presence of 1% sodium lactate and up to 1% NaCl. Growth occurred between pH 5.0–6.8. The strain showed tolerant only to rifamycin.

## Genome sequencing information

### Genome project history

Because it is one of the rare strains isolated directly from the soil, 10.1601/strainfinder?urlappend=%3Fid%3DNRRL+B-16219 strain was selected as part of an effort to gain insight into the depth of the pangenome pool and to identify symbiotic signaling molecules. The sequencing project was completed in April 2016 and the generated data was submitted as draft genome to Genbank under BioProject PRJNA318440 and the accession number MAXA00000000.1.

### Growth conditions and genomic DNA preparation

The studied strain was kindly provided by David Labeda, ARS USDA bacterial collection, as 10.1601/strainfinder?urlappend=%3Fid%3DNRRL+B-16219 strain ID. The strain was grown at 28 °C in stationary culture in 1-l bottles containing DPM medium [5], supplemented with 0.5 mM NH_4_Cl as nitrogen source maintained. Biomass from 1 month-old culture was harvested by centrifugation at 9000 x g for 15 min, rinsed several times with sterile distilled water. The mycelial mats were broken by repeated passages through syringes with progressively smaller diameters (21 g to 27 g). Genomic DNA extraction was performed using Plant DNeasy kits (Qiagen, Hilden, Germany) following the recommendation of the manufacturer. Prior to genome sequencing, the quality of the isolated DNA was checked by using the prepared DNA as template for PCR and partial sequences of several housekeeping genes and the 16S rRNA gene were generated and analyzed [16].

### Genome sequencing and assembly

Sequencing of the draft genome of 10.1601/nm.7635 sp. 10.1601/strainfinder?urlappend=%3Fid%3DNRRL+B-16219 was performed at the Hubbard Center for Genome Studies (University of New Hampshire, Durham, NH) using Illumina technology [38]. A standard Illumina shotgun library was constructed and sequenced using the Illumina HiSeq2500 platform with pair-end reads of 2 × 250 bp. The Illumina sequence data were trimmed by Trimmonatic version 0.32 [39], and assembled using Spades version 3.5 [40], and ALLPaths-LG version r52488 [41].

### Genome annotation

The genome was annotated via the NCBI Prokaryotic Genome Annotation Pipeline. Additionally nod gene prediction analysis was done within the Integrated Microbial Genomes-Expert Review system developed by the Joint Genome Institute, Walnut Creek, CA, USA [42] developed by the Joint Genome Institute, Walnut Creek, CA, USA, using similarity search tools. This whole-genome shotgun sequence has been deposited at DDBJ/EMBL/GenBank under the accession number MAXA00000000.1. The version described in this paper is the first version, MAXA00000000.1. A summary of the project information is shown in Table 2.

**Table 2:** Project information

MIGS ID	Property	Term
MIGS 31	Finishing quality	Draft genome
MIGS-28	Libraries used	Illumina Standard library
MIGS 29	Sequencing platforms	Illumina HiSeq2500 platform
MIGS 31.2	Fold coverage	120.5×
MIGS 30	Assemblers	Spades version 3.5, ALLPaths-LG version r52488
MIGS 32	Gene calling method	GeneMarkS+ v3.3
	Locus Tag	BBK14_RS02460
	Genbank ID	MAXA00000000.1
	Genbank Date of Release	October 30, 2016
	GOLD ID	Gp0153653
	BIOPROJECT	PRJNA224116
MIGS 13	Source Material Identifier	NRRL B-16219
	Project relevance	Agricultural

## Genome properties

The draft genome of 10.1601/nm.7635 10.1601/strainfinder?urlappend=%3Fid%3DNRRL+B-16219 consisted of 289 DNA contigs that correspond to estimated genome size of 8,032,739 bp and a GC content of 71.7%. The draft genome contained 6859 total genes, including 6211 protein-encoding genes (90.55%), 561 pseudo genes (8.17%) and 53 RNAs (0.76%) (Table 3). Classification of genes into the COG functional categories is shown in Table 4.

**Table 3:** Genome statistics

Attribute	Value	% of Total^a^
Genome size (bp)	8,032,739	100.0
DNA coding (bp)	6,603,166	82.20
DNA G + C (bp)	5,760,840	71.72
DNA Contigs289100		.0
Total genes	6859	100.0
Protein coding genes	6, 211	91.01
RNA genes	53	0.77
Pseudo genes^b^	561	8.18
Genes in internal clusters	-	-
Genes with function prediction	5046	73.60
Genes assigned to COGs	3609	52.64
Genes with Pfam domains	4735	69.06
Genes with signal peptides	176	2.57
Genes with transmembrane helices	296	4.32
CRISPR repeats	2	-

^a^The total is based on either the size of the genome in base pairs or the total genes in the annotated genome

^b^Pseudo genes may also be counted as protein coding or RNA genes, so is not additive under total gene count

**Table 4:** Number of genes associated with the general COG functional categories

Code	Value	% age^a^	Description
J	178	4.27	Translation, ribosomal structure and biogenesis
A	1	0.02	RNA processing and modification
K	408	9.79	Transcription
L	109	2.62	Replication, recombination and repair
B	1	0.02	Chromatin structure and dynamics
D	32	0.77	Cell cycle control, cell division, chromosome partitioning
V	135	3.24	Defense mechanisms
T	249	5.98	Signal transduction mechanisms
M	173	4.15	Cell wall/membrane biogenesis
N	21	0.5	Cell motility
U	30	0.72	Intracellular trafficking, secretion, and vesicular transport
O	140	3.36	Posttranslational modification, protein turnover, chaperones
C	250	6	Energy production and conversion
G	207	4.97	Carbohydrate transport and metabolism
E	297	7.13	Amino acid transport and metabolism
F	94	2.26	Nucleotide transport and metabolism
H	262	6.29	Coenzyme transport and metabolism
I	351	8.42	Lipid transport and metabolism
P	210	5.04	Inorganic ion transport and metabolism
Q	256	6.14	Secondary metabolites biosynthesis, transport and catabolism
R	508	12.19	General function prediction only
S	178	4.27	Function unknown
-	3247	47.36	Not in COGs

^a^The total is based on the total number of protein-coding genes in the genome

## Insights from the genome sequence

### Comparison of genomes from 10.1601/nm.7635 sp. 10.1601/strainfinder?urlappend=%3Fid%3DNRRL+B-16219 and other 10.1601/nm.7635 species

The 10.1601/nm.7635 sp. 10.1601/strainfinder?urlappend=%3Fid%3DNRRL+B-16219 genome was compared to all of the 10.1601/nm.7635 genomes available at NCBI genome database including seven 10.1601/nm.7635 species including 10.1601/nm.7636 *,* 10.1601/nm.29311, 10.1601/nm.29312, 10.1601/nm.29887, *“* 10.1601/nm.30356 *”*, 10.1601/nm.29990, and “*F. asymbiotica*”, two *Candidatus* Frankia and other 10.1601/nm.7635 sp. strains. As shown for other closely related strains from cluster 3, strain 10.1601/strainfinder?urlappend=%3Fid%3DNRRL+B-16219 has one of the largest genome sizes (8,032,739 bp) with a high GC content of 71.72%. Genes shown or suggested to be involved in the actinorhizal symbiosis were detected. Nitrogenase genes were organized into one operon: *nif*H-D-K-E-N-X-orf1-orf2-W-Z-B-U and a non-linked *nif*V gene. Genes encoding the hydrogenase subunits were clustered into two operons. Genes for two different types of truncated hemoglobins, HbN and HbO, were also present.

### Nodulation pathway

**Fig. 4:**
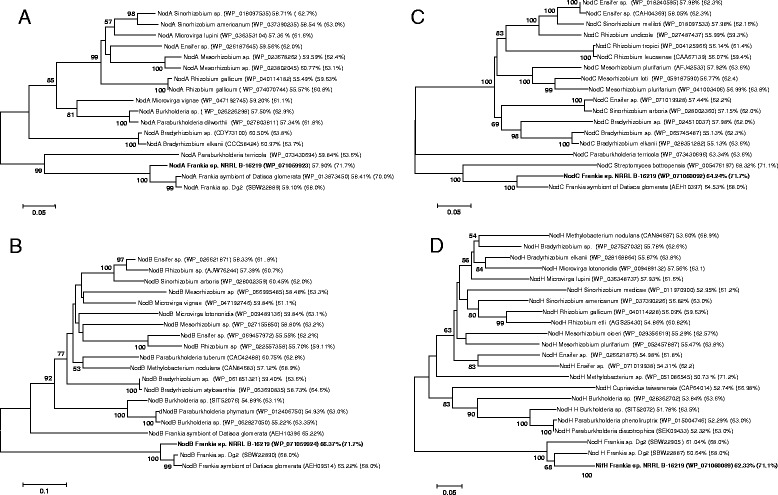
Maximum likelihood phylogeny based on amino acids of *nod*A (**a**), *nod*B (**b**), *nod*C (**c**) and *nod*H (**d**). GC-content is provided for *nod* genes and for genomes (in parenthesis). Bootstrap and probability values larger than 50% are only shown

In rhizobia, the common canonical *nod*ABC genes playing a key role in triggering root nodule formation in Legumes. These signals are secreted as a reply to host-plant flavonoids perceived by the compatible rhizobial strains [43]. The Nod factors perceived by host plant through the LysM-RLKs, and the resulting signal transduction cascade triggers a bacterial invasion of root cortical cells and the genesis of functional nodules. Despite the presence of these LysM-RLKs in the actinorhizal plants [11], none of the 10.1601/nm.7635 genomes from cultivated strains contained any homologous *nod* genes [33], but they are present in the two *Candidatus* Frankia genomes [32, 36]. Six *nod*-like genes were detected in the 10.1601/strainfinder?urlappend=%3Fid%3DNRRL+B-16219 draft genome (Additional file 1: Table S1) organized into two regions (Fig. 3). The first cluster contained genes encoding the *nod*A1, *nod*C, ABC-2 type transport system ATP-binding protein (*nod*J), ABC-2 transporter efflux protein, DrrB family (*nod*I) and *nod*H. The second cluster contained *nod*A, *nod*B and a *nod*H genes. Amino acid sequence similarities between 10.1601/nm.7635 sp. strain 10.1601/strainfinder?urlappend=%3Fid%3DNRRL+B-16219 NodA, B, C, and H predicted proteins ranged from 86 to 93% and 57–67% with the uncultured 10.1601/nm.7635 (Dg1 and Dg2) and (α- and β-) rhizobia, respectively (Additional file 2: Table S2). Further phylogenetic analysis (Fig. 4) showed that the 10.1601/nm.7635 Nod proteins were positioned at the root of both the α- and β-rhizobial NodABC proteins as previously reported [4, 8]. They were most closely related to plant nodulating 10.1601/nm.1616 of 10.1601/nm.1619 and 10.1601/nm.26956 genera. The GC content of 10.1601/nm.7635 *nod* genes ranged from 57.9% for *nod*A to 66.37% for *nod*B which is quite similar to that of some rhizobial species including 10.1601/nm.1581 and *Burkoldaria*. For both 10.1601/nm.7635 and rhizobia, GC% of the *nod* genes was lower than that of total genome sequences.

### Field collected microsymbionts of *Ceanothus americanus* contain *nod* genes

**Fig. 5:**
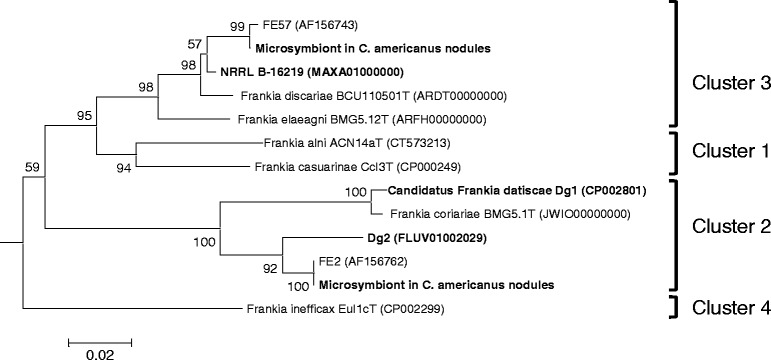
Neighbor-Joining phylogenetic tree based on *gln*A gene sequences. Bootstrap and probability values larger than 50% are only shown. Marked in *bold* are Frankia strains or microsymbionts with *nod* genes as present in their genomes or detected by PCR-sequencing analysis

Root nodules from *Alnus glutinosa*, *Casuarina glauca* and *Elaeagnus angustifolia* growing in Tunisia and *Ceanothus americanus* and *Elaeagnus umbellata* growing in Durham New Hampshire, USA, were collected. The *nod*A-*nod*B region from *C. americanus* nodules was PCR-amplified and sequenced. Following the alignments of the *nod*A and *nod*B gene sequences of Dg1 and 10.1601/strainfinder?urlappend=%3Fid%3DNRRL+B-16219, the primer set (forward primer nodAF 5′-AGCGCGACCCGAGCTCAGGATAATCG-3′ and reverse nodBF (5′-CGATCCCACCCGGATGGAGCTGC-3′) was designed in this study. The sequenced PCR-products were translated into amino acid sequences to permit the detection of the 23 aa sequence at the beginning of the 193 aa of the NodA, the intergenic region (160 nucleotides) and 41 aa at the end of the 230aa of the NodB. Both sequences showed 100% sequence similarities to their respective homologous region in NodA (23/193aa) and NodB (41/230aa) protein sequences for *Candidatus* Frankia Dg1. Regardless of their affiliation to cluster 2 or to cluster 3 (Fig. 5), all of the analyzed *C. americanus* microsymbionts contained the *nod*AB genes. In contrast, *A. glutinosa*, *C. glauca*, *E. umbellata* and *E. angustifolia* microsymbionts failed to amplify the expected PCR product. This result is in congruence with previous reports claiming that no homologous *nod* genes are retrievable in sequenced genomes from strains isolated from these actinorhizal plant species [33].

## Conclusions

We report here the genome sequence of a 10.1601/nm.7635 strain directly isolated from soil rhizosphere. The generated draft genome was assembled into 289 contigs corresponding to 8,032,739 bp, which falls within the size range of 10.1601/nm.7635 cluster 3 [33]. Bacterial factors triggering actinorhizal symbiosis remain enigmatic since many sequenced 10.1601/nm.7635 genomes have revealed the absence of universal nod-factors. It was hypothesized that most 10.1601/nm.7635 strains use a novel nod-independent pathway for the infection process of actinorhizal plants. In contrast, two *Candidatus* Frankia Dg1 and Dg2 genomes contain canonical nod genes [32, 36]. Here we provide the first proof for the presence of *nod* genes in the genome of a cultivated 10.1601/nm.7635 strain. In addition, a PCR-sequencing approach suggested that *nod* genes are only widespread in *C. americanus* microsymbionts. This situation is similar to legume symbionts where two nodulation pathways are described: the well-studied nod-dependent and an alternative nod-independent pathway. The majority of rhizobia use the nod-dependent pathway, while some photosynthetic [34] and non-photosynthetic [35] bradyrhizobia use the alternative nod-independent pathway. Moreover, some rhizobia use both pathways and the use of the nod-independent pathway seems to be highly dependent on host species rather than the presence or absence of *nod* genes in a given bradyrhizobial genome [44]. For 10.1601/nm.7635, almost all host plants are infected through the nod-independent pathway, while the nod-dependent process may only be present in unstudied actinorhizal species such as members of the genus *Ceanothus*.

## Additional files

Additional file 1: Table S1. Localizations and DNA coordinates for nod genes in NRRL B16219 and Dg1 genomes. (DOCX 12 kb)

Additional file 2: Table S2. Percent similarities based on amino acid sequence for NodA, B, C and H between *Frankia* sp. NRRL B-16219, *Candidatus* Frankia Dg1 and Dg2, and other rhizobial strains. (XLSX 308 kb)

## Abbreviations

DPM: Defined Propionate Medium
*gln*A: Glutamine synthetase
LysM-RLKs: LysM-receptor-like kinases
*nif*H: Nitrogenase reductase
*nod*A: Beta-1,4-N- acetylglucosamine oligosaccharide N-acyltransferase
*nod*A1: Beta-1,4-N-acetylglucosaminyltransferase
*nod*B: Beta-1,4-N-acettylglucosamine oligosaccharide deacylase
*nod*C: Beta-1,4-N-acetylglucosaminyltransferase
*nod*H: Beta-1,4-N-acetylglucosamine oligosaccharide 6-O-sulfotransferase
