# Supplementary material for: Permanent draft genome sequence of Frankia sp. NRRL B-16219 reveals the presence of canonical nod genes, which are highly homologous to those detected in Candidatus Frankia Dg1 genome
Source: Stand Genomic Sci. 2017 Sep 4;12:51. doi: 10.1186/s40793-017-0261-3 (PMC5584510; doi:10.1186/s40793-017-0261-3)
Supplement: Supplementary file 1 — Localizations and DNA coordinates for nod genes in NRRL B16219 and Dg1 genomes. (DOCX 12 kb) [file 40793_2017_261_MOESM1_ESM.docx]

**Table S1**: Localizations and DNA coordinates for nod genes in NRRL B16219 and Dg1 genomes

|  | NRRL B-16219  Locus Tag (DNA Coordinates) | Dg1  Locus Tag (DNA Coordinates) |
| --- | --- | --- |
| Beta-1,4-N-acetylglucosamine oligosaccharide 6-O-sulfotransferase **NodH**  ABC transporter efflux protein, DrrB family **NodI**  ABC-2 type transport system ATP-binding protein **NodJ**  Beta-1,4-N-acetylglucosaminyltransferase **NodC**  Chitooligosaccharide deacylase, NodA1 | BBK14_31585 (3203-3895)  BK14_31590 (4234-5169)  BBK14_31595 (5166-6140)  BBK14_31600 (6298-7617)  BBK14_31605 (7636-8145) | -  FsymDg_3087 (3665687-3666622)  FsymDg_3086 (3664716-3665690)  FsymDg_3085 (3663249-3664562)  FsymDg_3084 (3662361-3663230) |
| beta-1,4-N-acetylglucosamine oligosaccharide N-acyltransferase **NodA**  beta-1,4-N-acetylglucosamine oligosaccharide deacetylase **NodB (91.30)**  Beta-1,4-N-acetylglucosamine oligosaccharide 6-O-sulfotransferase **NodH** | BBK14_31085 (3037-3618)  BBK14_31090 (3778-4470)  BBK14_31110 (8347-8919) | FsymDg_2092 (2488619-2489200)  FsymDg_2093 (2489359-2490051)  - |
